# Supplementary material for: Complexome profiling of the Chlamydomonas psb28 mutant reveals TEF5 as an early PSII assembly factor
Source: Plant Cell. 2025 Mar 17;37(6):koaf055. doi: 10.1093/plcell/koaf055 (PMC12164590; doi:10.1093/plcell/koaf055)
Supplement: koaf055_Supplementary_Data [file koaf055_supplementary_data.zip › psb28_interactive.zip!/plots/intensity/qualityPlots/peptides/_Cre02.g143567.t1.1.html]

### Cre02.g143567.t1.1

Trivial name:   
SeqCoverage [median]: 25.30  
Mass: 17.91 kDa  
  
 

| Sequence | MissedCleavage | Mass [Da] | wtIntensity | muIntensity | Score |
| --- | --- | --- | --- | --- | --- |
| ALHELVRDD | 1 | 1066.54 | 4377.57 | 5635.78 | 119.68 |
| FTQITK | 0 | 736.41 | 2310.45 | 1761.09 | 120.49 |
| GVGYGALAGTALSTGLGAAK | 0 | 1733.93 | 2681.31 | 4768.69 | 119.21 |
| LHYNASQQR | 0 | 1115.55 | 2001.85 | 2285.31 | 103.19 |
|  |  |  | 11371.2 | 14450.9 |  |
